# A Rescue-Assistance Navigation Method by Using the Underground Location of WSN after Disasters †

**DOI:** 10.3390/s20082173

**Published:** 2020-04-11

**Authors:** Shuo Li, Tiancheng Guo, Ran Mo, Xiaoshuai Zhao, Feng Zhou, Weirong Liu, Jun Peng

**Affiliations:** 1 School of Electrical and Information Engineering, Changsha University of Science & Technology, Changsha 410114, China; guotiancheng0921@stu.csust.edu.cn (T.G.); zhoufengcsu@csust.edu.cn (F.Z.); 2 School of Computer Science and Engineering, Central South University, Changsha 410083, China; 0905130320@csu.edu.cn (R.M.); xiaoshuai@csu.edu.cn (X.Z.); frat@csu.edu.cn (W.L.); pengj@csu.edu.cn (J.P.)

**Keywords:** wireless sensor networks, indoor localization, guidance path, sparse anchor localization, disaster, anisotropic wireless sensor networks

## Abstract

A challenging rescue task for the underground disaster is to guide survivors in getting away from the dangerous area quickly. To address the issue, an escape guidance path developing method is proposed based on anisotropic underground wireless sensor networks under the condition of sparse anchor nodes. Firstly, a hybrid channel model was constructed to reflect the relationship between distance and receiving signal strength, which incorporates the underground complex communication characteristics, including the analytical ray wave guide model, the Shadowing effect, the tunnel size, and the penetration effect of obstacles. Secondly, a trustable anchor node selection algorithm with node movement detection is proposed, which solves the problem of high-precision node location in anisotropic networks with sparse anchor nodes after the disaster. Consequently, according to the node location and the obstacles, the optimal guidance path is developed by using the modified minimum spanning tree algorithm. Finally, the simulations in the 3D scene are conducted to verify the performance of the proposed method on the localization accuracy, guidance path effectiveness, and scalability.

## 1. Introduction

For underground disasters, the rescue operation is especially tricky due to the complex underground environment after a disaster occurring [1,2,3]. The underground disaster search and rescue, as the last line of defense for people trapped underground, are expected to find the escape or rescue paths quickly, precisely, and efficiently for the underground survivors as well as rescue staff. However, the traditional rescue paths may be generated based on outdated underground information by the ground rescue team [4,5], which cannot reflect the real scenarios in real-time. Nowadays, wireless sensor networks (WSNs) are widely deployed and play an increasingly important role in providing underground environment information [6]. The data collected by WSNs is critical in ensuring personnel safety, improving the ability of emergency rescue after disasters [7,8,9]. In practical disaster scenarios, the accuracy of node location is vital for generating the appropriate escape or rescue paths when using WSNs to assist the rescue mission [10]. In this paper, a guidance path developing method is proposed with the help of underground wireless sensor networks. In such a network scene, the distribution of network nodes is nonuniform due to the limitations of the underground building structure. This network is a typical anisotropic wireless sensor network. Especially after the disaster, the anisotropy of the network becomes more prominent. At the same time, disasters lead to a change in network topology and a sharp decrease in the number of reference anchor nodes. Therefore, the disaster rescue problem based on underground wireless sensor networks studied in this paper is essentially a location and path search problem for anisotropic networks with dynamic topology. The method proposed in this paper can also be used in similar scenarios, such as building disaster rescue.

When underground disasters occur, the underground topography will be changed greatly, causing unexpected collapses and fault belts. The tremendous change of underground topography deteriorates many communication links seriously and makes the communication among nodes under more complex and various conditions. Furthermore, the disaster will result in movements of most sensor nodes, and the original location information will not be in effect [11]. These nodes become so-called blind nodes. On the other hand, many nodes will become out of operation, which brings extreme difficulty for re-locating the precise positions of blind nodes, and thus, creates a challenge for generating the appropriate guidance paths. Thus, for the disaster scenario, it is important to address the issue that there are not sufficient trustable anchors close to the blind node, that is, the sparse anchor situation.

The node localization methods are classified into range-based methods [12] and range-free methods [13]. Generally, range-based methods can provide more accurate location information by using the distance measurement technologies, such as the angle of arrival (AoA), the received signal strength indication (RSSI) and the time of arrival (ToA), which both need to find the mechanisms transferring the measuring angles, strength or time to physical distances. For tiny wireless sensor nodes, it is the most common method to directly utilize the RSSI of the wireless signal without attaching other measurement modules. Nevertheless, this method depends on the accuracy channel model to transfer the RSSI to the distance between the source node and the destination node.

There are many works on the construction of the underground channel model. In Reference [14], a probabilistic sensing model for underground tunnels is utilized, but the multi-path fading effect is not considered in the channel model. In Reference [15], the shadowing effect is incorporated in the channel model by investigating radio wave attenuation, but the presence of obstacles and other factors are not considered. Thus, these channel models are not suitable for the scenario after disasters. In Reference [16], the Fisher information-based log-normal shadowing model is proposed, and the model can be used to analyze the existence of the collapsed hole. However, the analysis of the collapse obstacles cannot yet recur to the channel model. And the unexpected penetration of wireless signals should be incorporated in the channel model after disasters.

To address the obstacle effect, a semi-empirical mixing dielectric model is proposed for predicting the moist soil between sensor nodes [17], but the channel model has large errors for clay silt or sand soils. Consequently, a mineralogy based spectroscopic dielectric model is proposed to take into consideration the clay content [18]. Furthermore, the literature [19] derives a path loss model to describe the channel characteristics and propagation effects by taking the soil as a mixture of sand, silt, clay, and moisture. On the other hand, the literature [20] constructs the empirical model by considering the penetration loss when there are concrete brick walls among sensor nodes. Nevertheless, there are still short of the integrated channel model to comprehensively describe the complex underground communication environment. In this paper, a hybrid channel model is constructed by integrated different mixed kinds of communication models to present the appropriate features of underground wireless signals.

As for the range-based localization methods for the underground environment, in Reference [21], a chain-type network is presented which uses the low-density cluster structure, and the heads are deployed non-uniformly. A dynamic choice algorithm is proposed to improve the localization adaptability of the deployed sensor network. In Reference [22], an ultra-wideband based network is presented to provide the localization by combining different methods, including fingerprint-based geolocation system and Time-of-Arrival evaluation method.

Reference [23] proposes a localization system based on the two-tier placement of ZigBee anchors in underground mines. The anchors are placed optimally in the tunnels following either uniform or non-uniform placement strategy to reduce the energy consumption of sensors. In Reference [24], a novel localization approach is presented in the tunnels of underground mines by using the variants of real-time belief propagation to find the location of sensors and handled the non-Gaussian uncertainties. However, these works assume that the underground topologies are fixed and the tunnels are not partially destroyed. Nevertheless, it can not be neglected that the change topologies and the numerous invalid nodes in underground disasters.

One of the crucial problems is that the accidents will result in numerous nodes, including anchors and blind nodes, out of operation, which will bring the extreme difficulty for node localization. For improving the localization accuracy under the situation with insufficient anchors, a range-free localization algorithm called RAPS with trustable anchor pair selection for anisotropic networks is presented [25]. Similarly, Reference [26] presents an anchor selection algorithm called RAL, which picks the trustable anchors to address the sparse anchor problem. However, the localization algorithms proposed in References [25,26] are both based on the unique free-space communication model, which find it difficult to describe the complex underground communication scenarios. Thus, it is difficult for them to be directly applied to underground situations.

In this paper, firstly, the geological and spatial characteristics of the underground scenarios are analyzed. Then the hybrid channel model of communication is introduced for the underground scenario, which reflects the real characteristics of underground wireless communication. Based on the hybrid channel model of a complex underground environment, the localization method based on sparse anchors is proposed for emergent accidents. In the proposed method, the anchor selection algorithm is designed to obtain the trustable anchors. Then the guidance path can be developed with fewer obstacles or shorter distances. Thus, the loss of life and personal injury can be reduced in case of an emergency. The main contributions are summarized as follows:

- The hybrid channel model for underground scenarios is constructed, which integrates the analytical ray waveguide model, the fading effect of underground limit space, and the penetrating effect of obstacles. It can provide sufficient capacity to describe the communication characteristics of the complex underground environment, especially for disaster situations.
- The localization method based on anisotropic sparse anchors network after disasters is designed, which improves the accuracy of distance measurement by selecting the trustable anchors. Different sparse situations are analyzed, and the trustable anchors are chosen according to the hop count information, the distribution density of network nodes, and the theoretical minimum distance per hop.
- The optimal safety guided path is generated based on network node location information for the underground disaster scene. The spanning-tree based on network topology is constructed from the nodes in the trapped area to exit. All algorithms and propagation channel models are verified and tested in a 3D underground wireless sensor network scenario based on Matlab.

The remainder of this paper is organized as follows—Section 2 presents the hybrid channel model of underground communications. The proposed localization and navigation method is presented in Section 3. In Section 4, the performance of the proposed method is analyzed and verified by presenting numerous simulation results, followed by the conclusions and future works in Section 5. Table 1 summarizes the notations of this paper.

## 2. System Assumption and Channel Model

In this paper, if the previous deployed wireless network still keep the connections, it is feasible to develop the guidance path to accelerate the survivors escaping. The optimal escape guidance path can be designed according to the locations of nodes. However, after the disasters, many nodes will become blind nodes and need relocalization. If one node location does not change, and its saved location information is still valid, the node can be taken as the anchor to locate blind nodes. In order to achieve the objective of node localization and path developing, it is preliminary to make some assumptions for the scenario after disasters:

1. Although many nodes become invalid, the WSN previously deployed can still keep the network connectivity. The communication can be revived from the collapse area to the escape exit by node relay.
2. There are still some nodes that do not move and can know their accuracy locations. These nodes with valid location information can be taken as anchors to locate other blind nodes. Each anchor has its known accurate position and can share its information with other nodes, including the number of hops, multi-hop paths, position information, and RSSI to sensor nodes. The specific method for determining whether the node location information is valid is described in Section 3.1.
3. In some areas with sparse anchor distribution after disasters, there are not enough trustable anchors in 1-hop communication. Thus, it is necessary to find trustable anchors by multi-hop communication. For the worst case, some relocated nodes will be chosen as the anchors to locate the other blind nodes.
4. A gateway is set at each escape exit of the mine. The green square in the first picture of Section 4 is the gateway. The gateway is connected to the data aggregation center on the ground through a wired network (industrial Ethernet or fiber-optic network). Underground sensor nodes are connected to the gateway via wireless links.

**Figure 1:**
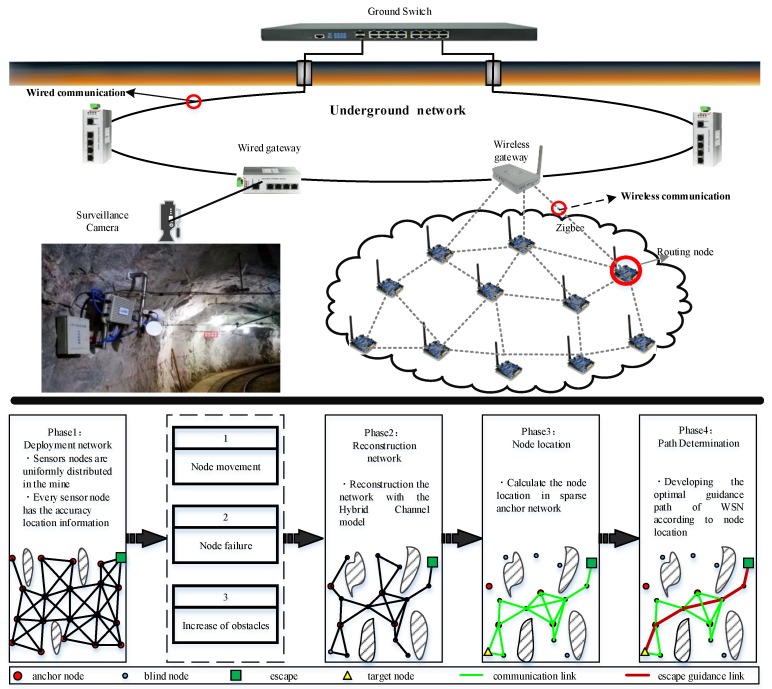
The framework of the guidance method for underground rescue.

Based on these assumptions, a node localization and path guidance method is proposed, which mainly includes four phases, as shown in Figure 1. The underground mine is composed of a large number of tunnel-like structures, and these structures are intertwined. It is a typical 3D network scene, which is characterized by complex terrain, narrow space, and the emergency treatment mechanism for accidents and disasters that must be considered. An emergency rescue communication system is deployed in such a scenario. The requirements for the communication system are that high bandwidth is not necessary, the network has a strong resilience and robust reconfiguration (self-organization) capabilities, and both communication capabilities and environmental awareness capabilities are essential. Therefore, it is appropriate to construct an emergency rescue system for underground mines based on wireless sensor networks.

A complete underground mine emergency rescue communication system is generally a mixed medium type (MMT) structure [27,28], including ground subnets and underground subnets. The ground subnet is generally based on the through the wire (TTW) mode, and the underground subnet is mainly based on the through the air (TTA) mode. The data exchange between the ground subnet and the underground subnet is achieved through gateways connected in TTW mode.

In phase 1, the wireless sensor network is deployed in the underground scenario initially. Nodes are uniformly distributed, and each node has valid preset location information. The protocol for wireless sensor networks is ZigBee. In order to improve the connectivity of the network, a mesh network topology is constructed so that the network layer can search for the best escape path. In our system, there are only two types of nodes: coordinator and router. The gateway, as shown in Figure 1, acts as a coordinator to achieve network organization and data aggregation.

After the disaster, numerous nodes will change their actual locations and become blind nodes. Simultaneously numerous nodes will be destroyed, and the network will reconstruction the communication connection, which is phase 2. In phase 3, the nodes with invalid location information will calculate the location with the assistance of the anchors. After re-estimating the node location, the escaping path will be developed according to the node location.

Before disasters, the underground scenario has mainly consisted of laneways and a few lobbies. The laneway is a typically long and narrow wireless communication channel. Moreover, the halls can be approximated by the free-space communication model. After disasters, such as collapse, wireless sensor nodes will change in the following aspects—(1) The random number of sensor nodes will become failure and can not participate in the network activity anymore; (2) The locations of many nodes will be changed; (3) There may be some unexpected obstacles among sensor nodes and the channel characteristics will be changed. The communication condition will become more complex. Therefore, it is a critical issue of how to model the wireless channel of the underground scenario and how to simplify the model [25].

### 2.1. Analyzing the Communication Characteristics

The detection of obstacles and communication characteristics requires the consideration of the possible disaster scenarios in the underground mines. There are three kinds of typical communication modes: (1) line-of-sight propagation in the tunnel with the reflection and refraction overlapped between the two sensor nodes; (2) When a disaster occurs, the tunnel will collapse or be squeezed, there may no be the line-of-sight propagation. Thus large scale fading and multipath effect exist to decline the signal transmission between two sensor nodes; (3) In some severe disasters, the wireless channel between sensor nodes can even be completely shadowed by collapsed obstacles. The radio signal has to penetrate the obstacle to achieve communication. Under this case, the wireless channel has distinctly different characteristics from the above two cases. Thus, in this paper, a data-driven method is designed to detect the case that the wireless channel is completely shadowed by obstacles. In the following content, the data-driven method is described in detail, then these communication characteristics of three modes are analyzed individually.

#### 2.1.1. Propagation Characteristic in Underground Mine

Wireless communication in underground mines is rather different from that in the air on the ground. In the underground mines, there are extensive long and narrow tunnels with various depth levels. The length and widths of the mine tunnels have an obvious impact on wireless communication. The propagation characteristics of radio waves in underground mines vary significantly on different terrestrial conditions, such as roughness of sidewalls, disorientation in orebody, curvature and bends, gallery dimension, and cross-cuts. To accurately describe the effect of the territorial space of underground mine on the propagation characteristics of radio waves, the multimode waveguide model is utilized as Equation (1) [29,30]:

(1) $$P_{rx}\left(x,y,z\right)=P_{tx}G_{tx}G_{rx}{\left(\frac{1}{E_{0}}\sum_{m,n}C_{m,n}\cdot E_{m,n}^{eigen}\left(x,y\right)\cdot e^{-(\alpha_{mn}+j\beta_{mn})\cdot z}\right)}^{2}.$$

where, $P_{rx}\left(x,y,z\right)$ is the received power of the destination node at location (x,y,z). x,y,z are the space coordinates of the destination node. $P_{tx}$ is the transmitting power of the source node. $G_{tx}$ is the gain of transmitting antenna and $G_{rx}$ is the gain of received antenna. $C_{m,n}$ is the mode intensity. $E_{m,n}^{eigen}$ is the electromagnetic (EM) field distribution. $E_{0}$ is field at the transmitting antenna. $\alpha_{mn}$ is the attenuation coefficient and $\beta_{mn}$ is the phase shift coefficient. The detail calculations of above parameters can be referred to [30]. However, Equation (1) is difficult to compute when there is an accumulative item. Thus, In a practical scenario, it is more common to use the analytical ray waveguide model to calculate the propagation losses in the underground tunnel [31]:

(2) $$\begin{array}{cc} l_{d} & =10log_{10}\left(\frac{P_{tx}}{P_{rx}}\right)\\ & =5\lambda r\left(\frac{1}{\omega^{2}}log_{10}\frac{1}{|R_{1}{|}^{2}}+\frac{1}{h^{2}}log_{10}\frac{1}{|R_{2}{|}^{2}}\right)+CL, \end{array}$$

where $R_{1}$ and $R_{2}$ are the reflection coefficients of the tunnel vertical and horizontal walls at the grazing angles $\phi_{1}=\lambda /2\omega$ and $\phi_{2}=\lambda /2h$, respectively. And ω is the width of the tunnel, *h* is the height of the tunnel, CL is the coupling losses of the antennas. The coupling loss for an antenna located at point (x,y) in the tunnel cross-section is derived in [31] as:

(3) $$CL=20log\left(\frac{2\pi \omega h}{\lambda^{2}G}cos^{-2}\left(\frac{\pi x}{\omega }\right)cos^{-2}\left(\frac{\pi y}{h}\right)\right).$$

where *G* is the gain of the antenna.

#### 2.1.2. Attenuation Characteristic by Shadowing Effect

When the signal is transmitted in the underground laneway, the reflection and refraction will occur due to the influence of the underground scenario wall and obstacles, which cause serious signal decay. Under this case, it is difficult and imprecise to analyze the attenuation effect of the practical environment factors and to construct the theoretical model based on the features of electromagnetic waves. Therefore, constructing the empirical model is usually an effective method. The statistical characteristics are summarized rather than analyzing the mechanism of the actual communication. According to the principle of minimum error, the mathematical relation forms of the parameters and variables are concluded. The empirical model only considers the relationship between input, output, and parameters, which is independent of communication mechanisms.

Numerous conducted experiments indicate that the mean path loss increases exponentially with distance. That is, the mean path loss is a function of distance. Thus, it is more visual to plot the power-distance relationship on the log-log scale, by which the relationship is a straight line. Absolute mean path loss, in decibels, is defined as the path loss from the transmitter to the reference distance $d_{0}$, plus the additional path loss in decibels. At this point, the log-normal propagation model is [32]:

(4) $$l_{d}=l_{0}+10N_{p}\log_{10}\frac{d}{d_{0}}+X_{\sigma },$$

where $X_{\sigma }$ is the Gaussian random variable with a zero mean. $N_{p}$ is the distance exponent, which is equal to 2 in free space. In the underground scenario Tunnel, signal propagation is similar to the indoor environment, and the value of $N_{p}$ can be set to great than 2. However, more accurate $N_{p}$ can be obtained by optimizing the propagation errors. $d_{0}$ is the reference range. $l_{0}$ is the path loss at the reference distance $d_{0}$. To obtain $l_{0}$ accurately, the millimeter-wave link budget model [33] is introduced to calculate the reference received power:

(5) $$l_{0}=-log_{10}[\frac{1}{NM}\sum_{i=1}^{N}\sum_{j=1}^{M}{\left|H_{j}\left(f_{i},d\right)\right|}^{2}],$$

where $H_{j}\left(f_{i},d\right)$ is the *j*th Tunnel transfer function at frequency $f_{i}$ and distance *d*. *N* is the number of sweep point in every transfer function and *M* is the number of measurements at each point. The detail calculations of above parameters can be referred to [33].

#### 2.1.3. Penetration Characteristic of Obstacle

When some collapse occurs in the underground environment, there will be obstacles between sensor nodes, and communication links between sensor nodes will be blocked. Because the Tunnel of the underground laneway and multi-floor Tunnel have similar characteristics, the model of path loss can be established by considering the space loss of Tunnel boundary absorption [25]:

(6) $$P_{rx}=P_{rx0}-10N_{p}\log_{10}\left(\frac{d}{d_{0}}\right)+X_{\sigma }-\left\{\begin{array}{cc} W_{a}\cdot F_{a} & W_{a}<C\\ C\cdot F_{a} & W_{a}\geq C \end{array}.\right.$$

Like the above model, *d* is the distance between the transmitting device and the receiving device. $d_{0}$ is the reference range. $P_{rx0}$ is the signal strength at the reference range $d_{0}$. *C* is the threshold value of the number of signals that pass-through obstacles. $W_{a}$ reflects the number of obstacles between the transmitting equipment and receiving equipment. Thus, it is a general model for multiple obstacles, in which the situation with just one obstacle is the special case with $W_{a}=1$. $F_{a}$ denotes the attenuation factor of a signal passing through the obstacle, which depends on the structure of the underground topology and the materials by which signal penetrated. Then considering the relationship between path loss and transmission power, there is [28]:

(7) $$l_{d}=P_{tx}+G_{tx}+G_{rx}-P_{rx}.$$

Taking (7) into (6), it is can be obtained as:

(8) $$l_{d}=l_{0}+10N_{p}\log\left(\frac{d}{d_{0}}\right)+X_{\sigma }+\left\{\begin{array}{cc} W_{a}\cdot F_{a} & W_{a}<C\\ C\cdot F_{a} & W_{a}\geq C. \end{array}\right.$$

After a radio signal radiates into the air through the antenna, its intensity will be attenuated due to the negative effects of absorption, transmission distance, and multi-path phenomenon. Different materials often produce different decay results. The 2.4 GHz signal drops 3 dB after penetrating the drywall, meaning that the amplitude decreases double. The 2.4 GHz signal penetrating the brick wall will decrease by 12 dB, which means the amplitude is 16 times lower than the original amplitude. Water and dense materials, including cinder bricks, are the main causes of radio absorption, all of which lead to signal decay greatly.

#### 2.1.4. Obstacle Detection Method Based on Data-Driven

When some collapse occurs in the underground environment, there will be obstacles between sensor nodes, and communication paths between sensor nodes will be blocked. In an indoor environment, only a single sampling and processing of the RSS of a sensor node cannot determine whether there are obstacles in the link between the nodes. However, with the receiver and transmitter positions determined, We can build a data set containing a large number of RSS samples. By extracting the feature of the data set and analyzing it, it is possible to construct a data-driven model to detect the presence of obstacles between links.

In the indoor environment, we set a pair of sensor nodes in the room and corridor, respectively, and use doors to simulate obstacles. Closing the door simulates the presence of obstacles between links. The transmitting node sends data in a cycle of 0.025 s, and the receiving node completes the sampling of the RSS samples once, and 1000 samples create a sample group. For every 100 sample groups collected, the location of the receiving node is changed and recorded. The final sample set is 1600 groups (800 groups with obstacles). All RSS samples are randomly divided into 5 Sample subsets for cross-validation. Considering the influence of the antenna direction on the experimental results, during the experiment, the transmitting node and the receiving node still ensure that the antennas are opposite and parallel even after redeployment.

**Figure 2:**
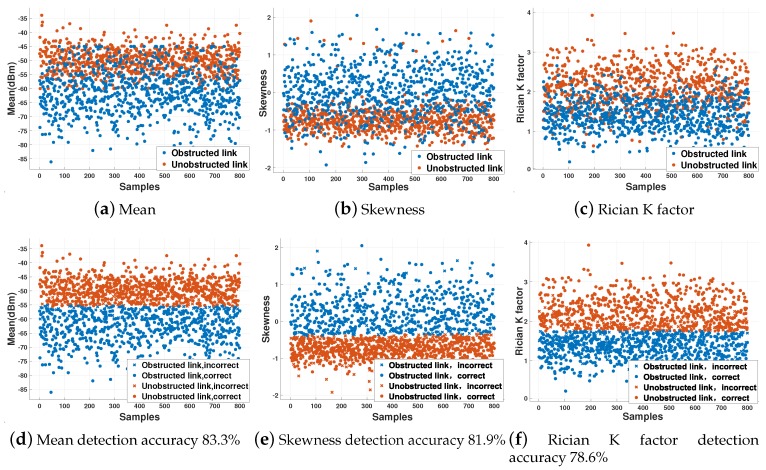
Illustration of how different features can detect the presence of the obstacles. (**a**–**c**) is the raw feature data without analysis. (**d**–**f**) is the result of processing the feature data using the hypothesis testing classifier (HTC) algorithm.

In this paper, a link obstacle detection algorithm based on hypothesis testing classifier (HTC) is adopted and compared with the support vector machine method. First, feature extraction is performed on the RSS samples. The extracted features include the mean, the skewness, and the Rician K Factor. Figure 2 is the result of analyzing the typical features of the sample set using the HTC algorithm. Figure 2a–c is the raw feature data without analysis. Figure 2d–f is the result of processing the feature data using the HTC algorithm. As can be seen from Figure 2d–f, an independent analysis of the three characteristics of the mean, the skewness, and the Rician K Factor can obtain 83.3%, 81.9%, and 78.6% accuracy of obstacle detection. By fusing the three features, the accuracy of detecting obstacles can reach 95%.

Compared with the Support Vector Machine (SVM) method, the HTC algorithm has slightly lower accuracy, but the training time is significantly reduced. Therefore, the HTC algorithm is more suitable for mobile computing and embedded systems. In order to achieve the obstacle detection between links, the likelihood ratio detection, including two competing hypotheses (with obstacles/without obstacles), is defined as

(9) $$\begin{array}{c} \begin{array}{c} T_{b}:t\leq t_{j},\;with\;obstacle\left(b=1\right),\\ T_{nb}:t>t_{j},\;without\;obstacle\left(b=-1\right). \end{array} \end{array}$$

where $t_{j}$ is the obstacle detection threshold and the value is 1. The definition of *t* is as

(10) $$t=\frac{p(x^{\left(1\right)},x^{\left(2\right)},x^{\left(3\right)}|T_{b})}{p(x^{\left(1\right)},x^{\left(2\right)},x^{\left(3\right)}|T_{nb})}=\prod_{i=1}^{3}\frac{p(x^{\left(i\right)}|T_{b})}{p(x^{\left(i\right)}|T_{nb})},$$

where $x^{\left(i\right)},i=1,2,3$ is the three features the mean, the skewness, and the Rician K Factor extracted from the RSS samples. $p(x^{\left(1\right)},x^{\left(2\right)},x^{\left(3\right)}|T_{b})$ and $p(x^{\left(1\right)},x^{\left(2\right)},x^{\left(3\right)}|T_{nb})$ are the joint distribution. In order to simplify the calculation, consider that the variables are independent. The definition of h is finally shown in the Equation (10), where $p(x^{\left(i\right)}|T_{b})$ and $p(x^{\left(i\right)}|T_{nb})$ are the distribution of features $x^{\left(i\right)}$ with and without obstacles, respectively. According to the analysis of the RSS samples, the distributions of features follow the Gaussian distribution.

**Figure 3:**
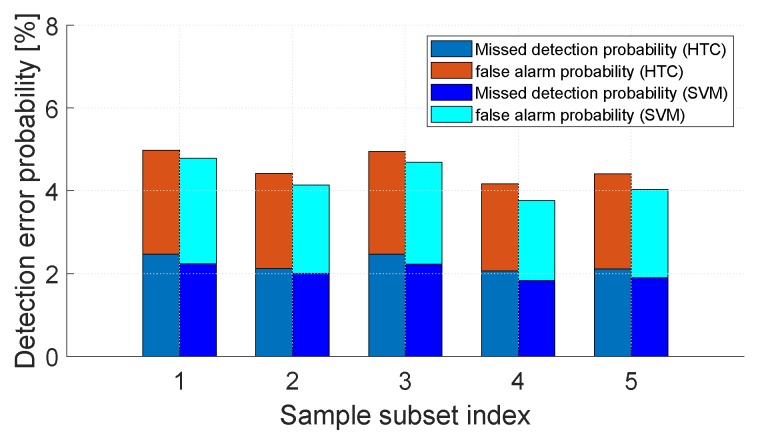
Missed detection probability, false alarm probability, and overall detection error probability of the HTC and SVM, showing the impact of different Sample subsets.

The performance of the HTC algorithm is evaluated in terms of missed detection probability (deciding without obstacles when the RSS samples are from with obstacles conditions) and false alarm probability (deciding with obstacles when the RSS samples are from without obstacles conditions). The overall detection error probability is the sum of the above two probabilities. The experimental results are shown in Figure 3, where the x-axis is the index of the Sample subset, and the y-axis is the detection error probability. It can be seen from the Figure 3 that for each Sample subset, the overall detection error rate of the HTC algorithm is 4.98%, 4.42%, 4.95%, 4.17%, 4.41%. The detection accuracy is close to or greater than 95%. It can be seen from the experimental results that the accuracy of the HTC algorithm is lower than that of the SVM algorithm. This is because the HTC algorithm is a linear classification algorithm, and the majority of the presence of obstacles between links is not linearly separable.

In summary, if a sample group contains 1000 samples and the frame sending period of the sensor node is 25 ms, the detection of whether the link is blocked by obstacles can be completed in 25 s, and the detection error is less than 5%.

### 2.2. Constructing the Hybrid Model

In the practical underground environment, especially after disaster occurs, the communication situation is complex. There may exist some adverse effects such as multipath effects, shadow effects, and obstacle blocking. Therefore, the unique model can not sufficiently describe the propagation characteristics of wireless signals in an underground mine environment.

**Figure 4:**
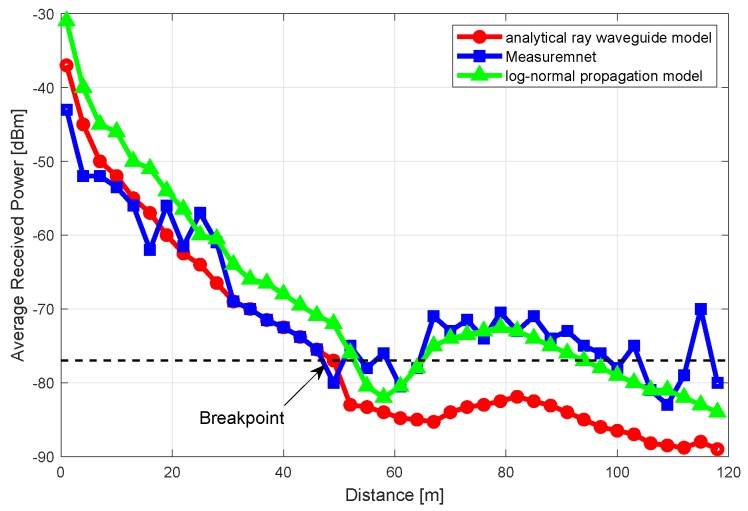
The measured and simulated power attenuation over distance in a tunnel.

This view was also confirmed by wireless signal propagation measurement experiments performed in a tunnel environment. The experiment uses the TelosB mote. During the experiment, the antenna of TelosB is kept as a vertical polarization pattern, and the gain is 2 dB. The experimental results are shown in Figure 4. It can be seen that different models have different approximation effects under different conditions. When the distance is less than 50 m, the analytical ray waveguide model has the best performance, while the distance is greater than 50 m, the log-normal propagation model has the best performance. To describe the underground communication model more accurately, the two classical models are integrated into one hybrid channel model.

In the initial assumption, every node has the accurate location information when deploying. In the case of the disaster, the nodes will deviate away from their initial location. First, the HTC algorithm described in Section 2.1.4 is used to determine whether there is an obstacle between the node links. If there are obstacles, the third sub-model of Equation (11) is used to estimate the distance between nodes. If no obstacle is detected, then based on the path loss, the analytical ray waveguide model and log-normal propagation model are selected for distance estimation.

However, the original locations can provide a rough estimation of path loss between nodes. If let $l_{f}$ denote the path loss calculated by using the original location and the free-space model, and let $l_{r}$ denote the actual measured path loss, the loss thresholds can be determined to select the sub-models of the hybrid model. Then the hybrid channel model incorporating the above cases to calculate the path loss can be described as:

(11) $$l_{d}=\left\{\begin{array}{cc} 5\lambda r\left(\frac{1}{\omega^{2}}log_{10}\frac{1}{|R_{1}{|}^{2}}+\frac{1}{h^{2}}log_{10}\frac{1}{|R_{2}{|}^{2}}\right)+CL & l_{r}\geq \eta_{1}l_{f}\\ l_{0}+10N_{p}\log_{10}\frac{d}{d_{0}}+X_{\sigma }\eta_{2} & \eta_{1}l_{f}>l_{r}\geq \eta_{2}l_{f}\\ l_{0}+10N_{p}\log\left(\frac{d}{d_{0}}\right)+X_{\sigma }+B\cdot F_{a} & l_{r}<\eta_{2}l_{f} \end{array},\right.$$

in which *B* = $W_{a}$, if $W_{a}$ < *C* and *B* = *C* otherwise. $\eta_{1}$ and $\eta_{2}$ are the model parameters used to select the piecewise sub-models, which correspond to the prior path loss $l_{f}$ between nodes before disasters, and can be set according to the practical experiments.

By utilizing the hybrid channel model, the distance between nodes and anchors can be measured. The hybrid channel model sufficiently describes the complex underground communication features. The sensor node senses the transmission losses of the closest anchors. Then judges the piecewise conditions of the hybrid model. Based on it, the model parameters are fitting by the attenuation or penetration of wireless signals. After determining parameters, the distance between the sensor node and the corresponding anchor can be calculated, which is necessary for the node locating and guidance path developing.

## 3. Node Localization and Guidance Path Method

This paper focused on developing an escape guidance path for underground survivors. The fundamental requirement of the guidance path is that the path can make the detour for obstacles with the shortest length. Obstacle avoidance can be achieved by utilizing the hybrid channel model to estimate the obstacle existence. The developing path with the shortest length should recur to the location of nodes. Just using the node location information, the node can pick out the node within 1-hop closest to the escape exit. However, after disasters, numerous node locations will change, and the initial location information will be invalid. In some cases, there are not sufficient anchors which still have location information. This brings the challenge for node relocalization. This section discusses node localization and guidance path method under the anisotropic sparse anchors network after disasters.

### 3.1. Detection of Nodes Movement

In this paper, the method of wall contactor and accelerometer is used to judge the movement of nodes. Compared with the method of the accelerometer, the wall contactor method is simpler and lower cost, but the scalability and detection accuracy are relatively low.

#### 3.1.1. Method 1 Wall Contactor

**Figure 5:**
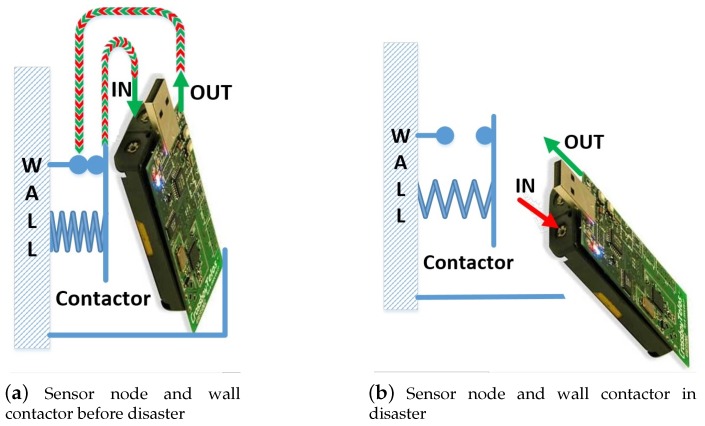
Illustration of detecting node movement with the wall contactor: (**a**) is a schematic diagram of the state of the sensor node and contactor before the disaster; (**b**) is a schematic diagram of the state of sensor nodes and contactors when a disaster occurs.

The ground contactor is a contact switch. As shown in Figure 5, the contactor is mounted on the wall and contains a pair of contacts and a spring-driven reset device. In the network deployment stage, the sensor node is placed in the contactor, the spring of the contactor is compressed, and the two contacts are closed to make the movement detection circuit of the sensor node conductive. When a disaster occurs, as shown in Figure 5b, due to shock and vibration, the sensor node is disengaged from the contactor, and the movement detection circuit of the sensor node is cut off. A real-time monitoring program is added to the software of the sensor node, and the state of the motion detection circuit can be monitored to determine whether the sensor node becomes a blind node due to a change in position.

#### 3.1.2. Method 2 Accelerometer

**Figure 6:**
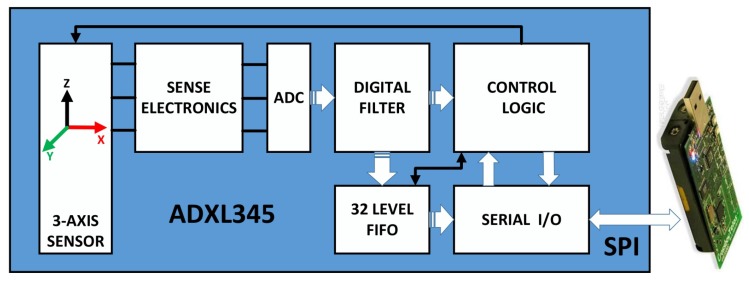
System block diagram of a sensor node with accelerometer installed.

As shown in Figure 6, the ADXL345 is a 3-axis accelerometer with 13bit resolution measurement at up to ±16 g. The ADXL345 is connected to the microprocessor of the sensor node via the SPI bus. The microprocessor receives data through the SPI bus and obtains three acceleration information in the vertical direction, which is used to calculate the sum magnitude vector (SMV) [34], as shown in Equation (12).

(12) $$SMV=\sqrt{A_{x}^{2}+A_{y}^{2}+A_{z}^{2}}$$

In the normal state, the sensor node is at rest, and the *SMV* is only affected by the acceleration of gravity. When a sensor node falls due to a disaster, the node is in a weightless state before landing, and the *SMV* value tends to 0. At the moment of landing, the node receives a shock, and the *SMV* value will increase instantly. By detecting this change process of the *SMV*, it can be judged whether the position of the sensor node changes and becomes a blind node.

### 3.2. Node Localization Algorithm

In the wireless sensor network, if there are sufficient anchors within the 1-hop range of a blind node, the trilateration localization algorithm can be exerted directly to estimate the position of the blind node with the range-based method. However, for underground scenarios after the disaster, because lots of sensor nodes become blind or invalid, there are not sufficient 1-hop anchors. So the remote anchors should be selected to participate in the localization, but for a blind node, not all remote anchor nodes are suitable localization reference nodes. These suitable positioning reference nodes are defined as trustable anchor nodes. In the following, the trustable anchor selection algorithm will be presented by analyzing different sparse cases.

**Figure 7:**
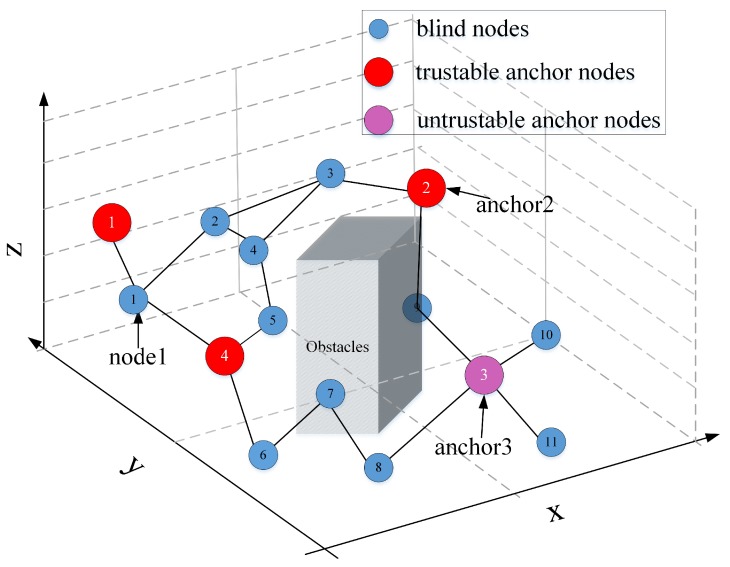
The example of trustable anchor judgment.

As shown in Figure 7, there are only two anchors within the 1-hop range of node 1. Thus the location of node 1 cannot be directly estimated. Then node 1 needs to find a remote anchor outside its 1-hop range as a location reference. However, if there are obstacles in the wireless network, not all remote anchors can be used as location references. In Figure 7, anchor 2 and anchor 3 are both remote anchor node to node 1. Nevertheless, because of the obstacle existence, the actual distance between anchor 3 and node 1 is much less than their estimated distance, so anchor 3 is taken as the un-trustable anchor for node 1. On the other hand, because the actual distance between anchor 2 and node 1 is approximate to their estimated distance, then anchor 2 can be taken as the trustable anchor for node 1.

Generally, in wireless sensor networks with obstacles and sparse anchors, the key problem is to judge whether the remote anchors are trustable. The basic idea of this paper is to calculate the shortest average distance per hop between the blind node *X* and the remote anchor *A* ($hl_{shortest}$) based on the current positioning information. Then the theoretical minimum distance per hop ($hl_{min}$) is calculated according to the node distribution density and the number of hops of the remote anchor. When $hl_{short}$ is less than or equal to $hl_{min}$, there is likely an obstacle between the blind node and the remote anchor node. Then remote anchor A is taken as an un-trustable anchor node; or else, the remote anchor A is taken as the trustable anchor.

#### 3.2.1. The Calculation of Theoretical Minimum Distance per Hop

In this paper, the theoretical minimum distance per hop is selected as the threshold for the differentiation of the trustable anchors. The $hl_{min}$ of nodes depends on the average distance per hop and the number of hops between nodes. Where the density of neighbor nodes determines the average distance per hop, and it can be calculated as:

(13) $$hl=r\left(1+e^{-\rho }-\int_{-1}^{1}e^{-\frac{\rho }{\pi }\left(\arccos t-t\sqrt{1-t^{2}}\right)}dt\right).$$

**Figure 8:**
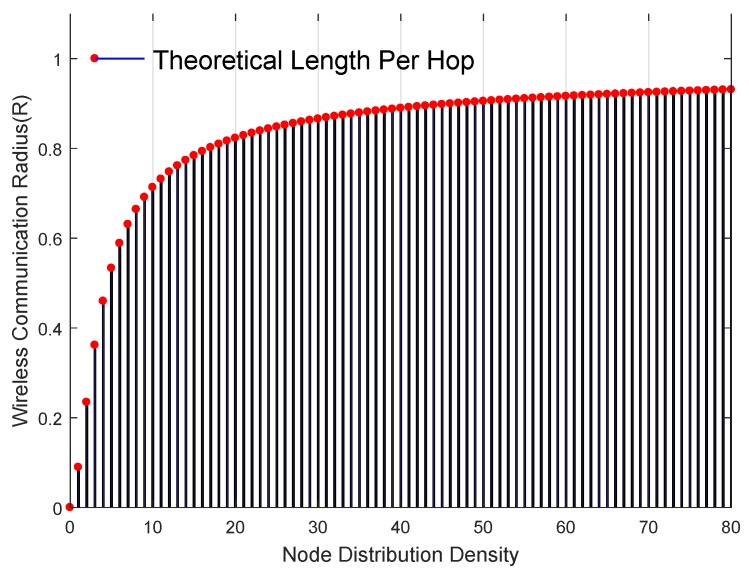
The average distance per hop for different densities of nodes.

According to (13), when the density of neighbor nodes increases, the average distance per hop also increases. It can be assumed that the node density ρ≥6 when the wireless network is deployed because the node density ρ=6 is the optimal value to ensure the network connectivity [28]. When the density of nodes is too high, the limit value of hl is communication radius *R*. Thus the maximum distance per hop is *R*. Figure 8 illustrates the relationship between the theoretical average distance per hop and the wireless communication radius under different node distribution density.

Then the theoretical minimum distance per hop $hl_{min}$ is calculated as following: when the number of hop h=1, the blind node may be infinitely close to the anchor. In this case, the real minimum distance between the anchor and node, $pl_{min}=0$, and we have $hl_{min}=pl_{min}/h=0$; When the number of hops h=2, the position of the blind node may be infinitely close to the spherical face with the anchor node as the center and the communication range as the radius. Thus $pl_{min}=R$, and we have $hl_{min}=pl_{min}/h=R/2$. when h=3, the real minimum distance $pl_{min}=R+hl$, thus $hl_{min}=pl_{min}/h=\left(r+hl\right)/2$. Similarly, when h=k, $hl_{min}=pl_{min}/h=\left(R+\left(k-2\right)hl\right)/2\left(k\geq 3\right)$. The calculation can be summarized as (14):

(14) $$\left\{\begin{array}{c} h=1,hl_{min}=0;\\ h=k,hl_{min}=\frac{R+\left(k-2\right)hl}{k}\left(k\geq 2\right). \end{array}\right.$$

#### 3.2.2. The Calculation of Possible Minimum Distance per Hop

In this paper, the shortest possible minimum distance between blind nodes and anchors $hl_{shortest}$ is calculated considering the different number of neighbor anchors. When $hl_{shortest}\geq hl_{min}$, the remote anchor can be taken as trustable anchor. The trustable anchor selection is divided into the following three cases.

**Figure 9:**
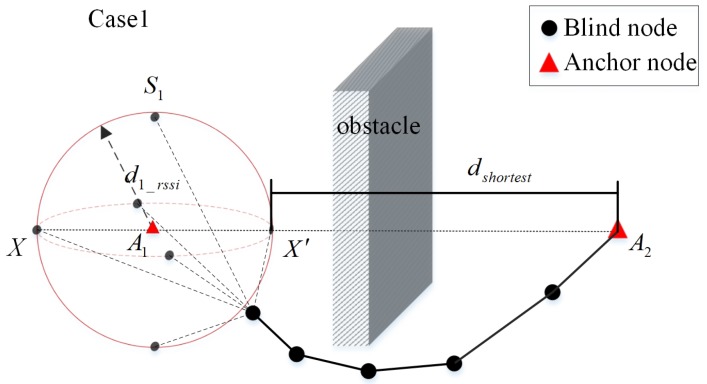
Trustable anchor node selection scenario for single neighbor anchor node.

When there is just one trustable anchor in the neighbor set of the blind node, the shortest possible distance between the blind node and the possible anchor is determined, as shown in Figure 9. $A_{1}$ is the trustable neighboring anchor of the blind node *X*, and the distance between the trustable anchor $A_{1}$ and the blind node *X*, $d_{1\_rssi}$ can be obtained by using the RSSI. Then, it can be assumed that the blind node *X* is located on the spherical face $S_{1}$ with the anchor node $A_{1}$ as the center and $d_{1\_rssi}$ as the radius.

To determine whether the possible anchor $A_{2}$ is a trustable anchor for the blind node *X*, the point, denoted by $X{}^{\prime }$, that has the shortest distance $d_{shortest}$ to the anchor $A_{2}$ can be found on the spherical face $S_{1}$. According to

$$hl_{shortest}=d_{shortest}/h,$$

the shortest average distance per hop $hl_{shortest}$ can be calculated. When $hl_{shortest}>hl_{min}$, the anchor $A_{2}$ can be taken as a trustable anchor for the blind node *X*.

**Figure 10:**
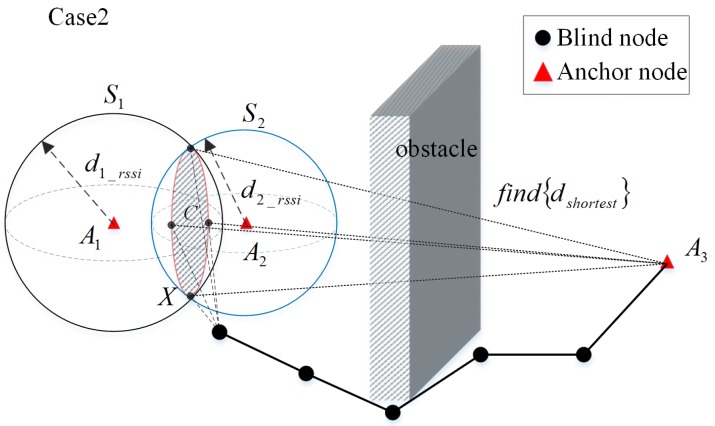
Trustable anchor node selection scenario for dual neighbor anchor nodes.

When there are two trustable anchors in the neighbor set of the blind node, the situation is shown in Figure 10. The anchor node $A_{1}$ and $A_{2}$ are the neighboring anchors of the blind node *X*. According to the hybrid model in the Section 2, the distances of the anchors $A_{1}$ and $A_{2}$ to the blind node *X* $d_{1\_rssi}$ and $d_{2\_rssi}$ can be obtained by measuring their RSSI respectively. It can be assumed that the position of the blind node *X* is on the circle *C* where the spherical face $S_{1}$ intersects the spherical face $S_{2}$. Determine the shortest distance $d_{shortest}$ between circle *C* and anchor node $A_{3}$. Thus, when $hl_{shortest}>hl_{min}$, the anchor $A_{3}$ can be taken as a trustable anchor for the blind node *X*.

**Figure 11:**
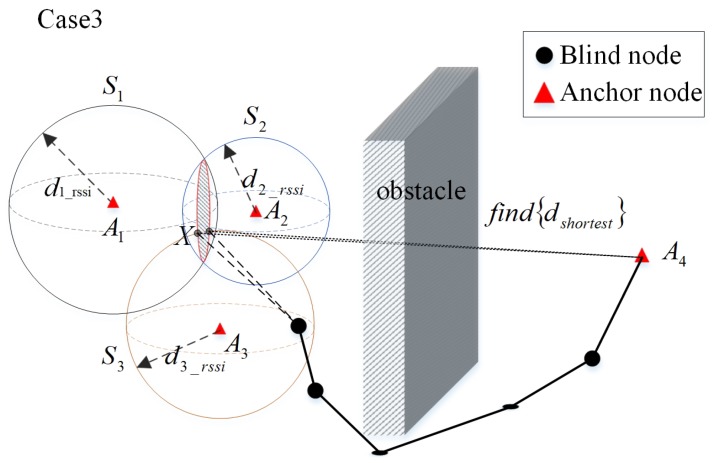
Trustable anchor node selection scenario for treble neighbor anchor nodes.

When there are three trustable anchors in the neighbor set of the blind node, the situation is shown in Figure 11. The anchor node $A_{1}$, $A_{2}$ and $A_{3}$ are the neighboring anchors of the blind node *X*. According to the hybrid model in the Section 2, the distances of the anchors $A_{1}$, $A_{2}$ and $A_{3}$ to the blind node *X* $d_{1\_rssi}$, $d_{2\_rssi}$ and $d_{3\_rssi}$ can be obtained by measuring their RSSI respectively. It can be assumed that the position of the blind node *X* is one of the two intersections of the spherical face $S_{1}$, $S_{2}$ and $S_{3}$. Let $d_{1}$ and $d_{2}$ denote the distance from the blind node *X* to the possible anchor $A_{4}$ respectively. Then the shortest distance, $d_{shortest}=min\left\{d_{1},d_{2}\right\}$. Thus, when $hl_{shortest}=min\left\{d_{1},d_{2}\right\}/h>hl_{min}$, the anchor $A_{4}$ can be taken as a trustable anchor for the blind node *X*.

#### 3.2.3. The Node Location by Using Trustable Anchors

In the 3D space scenario discussed in this paper, the trustable anchor node and its coordinates are denoted as $R_{1}$ $\left(x_{1},y_{1},z_{1}\right)$, $R_{2}$ $\left(x_{2},y_{2},z_{2}\right)$, … $R_{i}$ $\left(x_{i},y_{i},z_{i}\right)$. When the blind node x has more than four trustable anchor nodes, the coordinates of the blind node $X_{n}$ $\left(x_{n},y_{n},z_{n}\right)$ are theoretically estimated by solving Equations (15).

(15) $$\left\{\begin{array}{c} d_{1}^{2}={\left(x_{1}-x_{n}\right)}^{2}+{\left(y_{1}-y_{n}\right)}^{2}+{\left(z_{1}-z_{n}\right)}^{2}\\ d_{2}^{2}={\left(x_{2}-x_{n}\right)}^{2}+{\left(y_{2}-y_{n}\right)}^{2}+{\left(z_{2}-z_{n}\right)}^{2}\\ \vdots \\ d_{i}^{2}={\left(x_{i}-x_{n}\right)}^{2}+{\left(y_{i}-y_{n}\right)}^{2}+{\left(z_{i}-z_{n}\right)}^{2} \end{array}\right.$$

However, in a real wireless sensor network, whether using the range-based or range-free method, there is a deviation between the estimated distance between the nodes from the actual distance. The least-square method is an effective method to solve this problem. After decomposing and recombining the Equation (15), the new linear Equation (16) is obtained, and the matrix form of Equation (16) is shown in Equation (17).

(16) $$\left\{\begin{array}{c} d_{1}^{2}-d_{i}^{2}=x_{1}^{2}-x_{i}^{2}-2x_{n}\left(x_{1}-x_{i}\right)+y_{1}^{2}-y_{i}^{2}-2y_{n}\left(y_{1}-y_{i}\right)+z_{1}^{2}-z_{i}^{2}-2z_{n}\left(z_{1}-z_{i}\right)\\ d_{2}^{2}-d_{i}^{2}=x_{2}^{2}-x_{i}^{2}-2x_{n}\left(x_{2}-x_{i}\right)+y_{2}^{2}-y_{i}^{2}-2y_{n}\left(y_{2}-y_{i}\right)+z_{2}^{2}-z_{i}^{2}-2z_{n}\left(z_{2}-z_{i}\right)\\ \vdots \\ d_{i-1}^{2}-d_{i}^{2}=x_{i-1}^{2}-xi^{2}-2x_{n}\left(x_{i-1}-x_{i}\right)+y_{i-1}^{2}-y_{i}^{2}-2y_{n}\left(y_{i-1}-y_{i}\right)+z_{i-1}^{2}-z_{i}^{2}-2z_{n}\left(z_{i-1}-z_{i}\right). \end{array}\right.$$

(17) AX=B

Vectors X and Matrix A,B are shown in Equations (18)–(20), respectively.

(18) $$X={\left(\begin{array}{ccc} x_{n} & y_{n} & z_{n} \end{array}\right)}^{T}$$

(19) $$A=\left(\begin{array}{ccc} 2(x_{1}-x_{i}) & 2(y_{1}-y_{i}) & 2(z_{1}-z_{i})\\ 2(x_{2}-x_{i}) & 2(y_{2}-y_{i}) & 2(z_{2}-z_{i})\\ \vdots & \vdots & \vdots \\ 2(x_{i-1}-x_{i}) & 2(y_{i-1}-y_{i}) & 2(z_{i-1}-z_{i}) \end{array}\right)$$

(20) $$B=\left(\begin{array}{c} x_{1}^{2}-x_{i}^{2}+y_{1}^{2}-y_{i}^{2}+z_{1}^{2}-z_{i}^{2}+d_{i}^{2}-d_{1}^{2}\\ x_{2}^{2}-x_{i}^{2}+y_{2}^{2}-y_{i}^{2}+z_{2}^{2}-z_{i}^{2}+d_{i}^{2}-d_{2}^{2}\\ \vdots \\ x_{i-1}^{2}-x_{i}^{2}+y_{i-1}^{2}-y_{i}^{2}+z_{i-1}^{2}-z_{i}^{2}+d_{i}^{2}-d_{i-1}^{2} \end{array}\right).$$

Using the least-squares algorithm and solving the matrix equation to get the vector X, the best coordinates (*x*, *y*, *z*) of the anchor node *x* can be obtained. The simulations show that the greater the number of trustable anchor nodes, the higher the accuracy of blind node position estimation.

(21) $$X={\left(A^{T}A\right)}^{-1}A^{T}B.$$

### 3.3. Rescue Guidance Path Developing

When developing the escape path, generally, the shortest path can let the trapped people escape as quickly as possible. Thus, the algorithm is proposed to obtain the path with the shortest distance to the exit in all possible paths. The proposed algorithm is based on the minimal spanning tree which can find the path with the shortest distance between any two connected end nodes, called the source node $s_{s}$ and the target node $s_{t}$, by using the recursive process. The target node is the node closest to the exit. The difference between the proposed algorithm and the conventional minimal spanning tree algorithm is the setting of the branch weights of the tree. In the proposed algorithm, the branch weights of the tree integrate the distance factor and obstacle factor.

Similar to [7], the algorithm needs to research all possible paths in which the recursive process starts from the target node. Nevertheless, in this paper, different to Reference [35], the optimal path is determined by the actual measured distance of nodes according to node location, rather than the average distance estimated by the number of hops.

Firstly, the last hop nodes are found backward from the target node $s_{t}$, and then each node seeks its last hop nodes in the searching process until the source node $s_{s}$ is found. If there are no nodes near the current node, the algorithm will backtrack the preceding node deriving the current node to continue the searching process. When the algorithm finds a valid path, it stores the path by vector and calculates the distance of the valid path from the source node to target node hop by hop. Finally, all found paths are saved into the matrix $P_{st}^{h}$, and their distances are saved into the matrix $D_{st}^{h}$.

As shown in Algorithm 1, after researching all possible paths and calculating all path lengths, the shortest distance path can be selected. The shortest distance path can be recommended as the optimal escape paths. The computing complexity of Algorithm 1 is similar to the conventional minimal spanning tree constructing algorithm, so it is O($N^{2}$).
**Algorithm** **1** Algorithm description of escape guidance path developing1: **Input**: $s_{t}$2: **Output**: $P_{st}^{h}$3: **Initialize**: *h* = 1, node *i* = target node, node *u* = target node4: **while** (node *i* != source node) **do**5:  **if** node *i* has next trustable anchor node *n*
**then**6:   calculate the distance *d* between node *i* and node n7:   add the path $p_{h}$ to $P_{st}^{h}$8:   *h* = *h* + 19:   node *u* = node *i*, node *i* = node *n*10:  **else**11:    node *i* = node *u*12:  **end if**13: **end while**14: **for**
*j* = 1 to *h*
**do**15:  sum all *d* in $p_{h}$, calculate result $d_{sum}$16:  choose the shortest $d_{sum}$ as path $p_{h}$17: **end for**18: **return**
$P_{st}^{h}$

## 4. Simulation and Analysis

To validate the proposed node localization and guidance path method, the simulation for 3D underground scenarios are conducted. The node and obstacle distributions are illustrated before disasters as well as after disasters. In the simulation, a visualization program is designed, through which the performance of the trustable anchor node selection algorithm can be evaluated and tested intuitively in various network scenarios. Then the estimated locations of nodes are compared with their actual locations on the underground network scenarios. Further, the scalability of the proposed method is verified by adjusting the distribution density of the network node and the number of anchors. The simulations are conducted by MATLAB, in which the ZigBee protocol is simulated for node communication.

### 4.1. The Simulation Scenario and Parameter Setting

**Figure 12:**
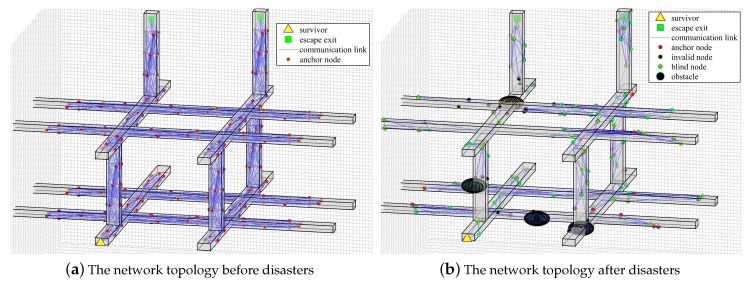
The network topology before and after disasters: (**a**) is the network topology before disasters; (**b**) is the network topology after disasters.

The key parameters of the simulation are shown in Table 2. Before the disaster, as shown in Figure 12a, all sensor nodes know their accurate position information and each node can be taken as the anchor. But after the disaster, as shown in Figure 12b, there appear many obstacles in the scenario which can affect wireless communication. Many nodes have been changed in their location and need to be relocated, and these nodes are called blind nodes. Some nodes have failed because they receive an impact, resulting in complete failure, and these nodes are called invalid nodes. The above effects make the network reorganize, and the network topology is also reconstructed.

### 4.2. Performance Evaluation of Trustable Anchor Node Selection Algorithm

In the location problem of anisotropic wireless sensor networks, the rationality of anchor node selection has a great influence on the location accuracy. To test the performance of the trustable anchor node selection algorithm proposed in this paper, and to intuitively reflect the factors affecting the correctness of the trustable anchor node selection, a visual anchor node selection evaluation program was designed.

After the initialization of the simulation scenario is completed, according to the global information of the network, the program counts the true number of anchor nodes and the anchor node ID of each blind node. After the simulation is completed, the program counts the number of suitable anchor nodes and the anchor node ID calculated by each blind node using the trustable anchor node selection algorithm. The visualization program compares the results of the two statistics and outputs a suitable anchor node selection accuracy rate as an evaluation of the performance of the trustable anchor node selection algorithm. Through simulation tests on 1000 groups of different network scenarios, the results show that the average correct rate of trustable anchor node selection is 90.3%.

Another function of the visualization program is to draw a 3D fitted surface based on the x-y coordinate information of the blind node and the number of misselected trustable anchor nodes. Through this 3d surface, the influence of network deployment on the correct selection rate of trustable anchor nodes can be intuitively analyzed.

**Figure 13:**
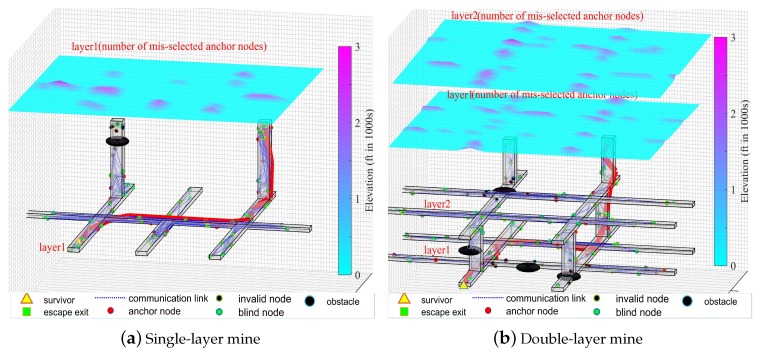
The performance evaluation of trustable anchor node selection algorithm: (**a**) is the visualization result of performance evaluation of the selection of trustable anchor nodes in single-layer scenarios; (**b**) is the visualization result of performance evaluation of the selection of trustable anchor nodes in double-layer scenarios.

Figure 13a,b are the simulation results of a single-layer and double-layer underground mine network, respectively. The lower part of the figure is the visualization of the network scene, and the upper part is the fitting surface of the number of misselected trustable anchor nodes. The simulation results show that the smoother the surface in the graph, the better the performance of the proposed anchor selection algorithm. From Figure 13, it can be observed that the correctness of the trustable anchor node selection of the proposed algorithm is related to the node distribution density. In areas with high node density, the correct rate of trustable anchor node selection is also high. There may be selection errors in areas where the node distribution density is low, especially in the network edge area.

### 4.3. Performance Evaluation and Comparison of Localization Algorithm

To present the scalability of the proposed method and give the quantifiable outcomes, the normalized root mean square error (NRMSE) is taken as the evaluation index, whose definition is Equation (22). Where the *m* is the number of blind nodes, the ${x^{\prime }}_{n},{y^{\prime }}_{n},{z^{\prime }}_{n}$ is the estimated coordinate of the blind node *n*, the $x_{n},y_{n},z_{n}$ is the actual coordinate of the blind node *n*, and the *R* is the transmission range.

(22) $$NRMSE=\frac{\sum_{n=1}^{m}\sqrt{{\left(x_{n}-{x^{\prime }}_{n}\right)}^{2}+{\left(y_{n}-{y^{\prime }}_{n}\right)}^{2}+{\left(z_{n}-{z^{\prime }}_{n}\right)}^{2}}}{mR}.$$

The three algorithms RAL [26], DV-distance [36] and CVLR [37] are selected as the comparison algorithms for the method proposed in this paper. The work in these papers is aimed at the problem of node localization in anisotropic wireless sensor networks.

- The algorithm RAL proposes an accurate average hop-length estimation algorithm. The algorithm can both tolerate irregular radio propagation and the distortion effect of obstacles, thus improving the node localization performance in anisotropic wireless sensor networks.
- The algorithm DV-distance proposes an assistant anchor localization strategy to solve the problem of low node localization accuracy in irregular (anisotropic) wireless sensor networks.
- The algorithm CVLR solves the LAEP’s hop-distance ambiguity problem based on position correction vectors, thereby improving the localization accuracy of blind nodes.

**Figure 14:**
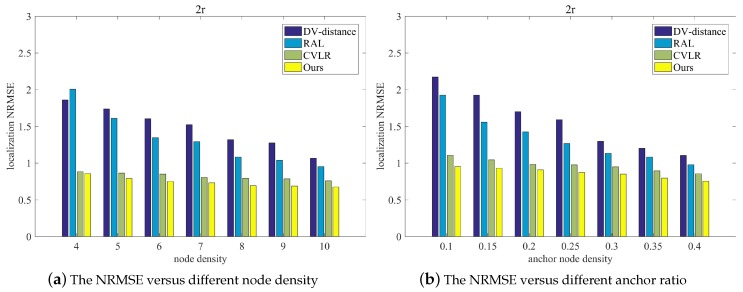
The normalized root mean square error (NRMSE) comparison for four algorithms under different node density and different anchor ratio: (**a**) is NRMSE versus different node density; (**b**) is NRMSE versus different anchor ratio.

To show the scalability of the proposed method, Figure 14a illustrates the NRMSE of the compared methods for different node density under the same anchor node ratio, and Figure 14b illustrates NRMSE comparison for methods under the different ratio of anchor nodes. From Figure 14a, it can be seen that with the increase of the node density, NRMSEs of other methods exhibit the same decrease trend, especially for the DV-distance and the RAL algorithm. While for the proposed method, there is not a decreasing trend because its iterations will increase with the number of blind nodes, and the localization accuracy cannot be improved. Nevertheless, the NRMSE of the proposed method is always the lowest compared to other methods.

From Figure 14b, it can be seen that with the increase of the ratio of anchor nodes, all NRMSEs of different algorithms exhibit the decreasing trend. It is reasonable that the increase in the number of anchors will let the blind node have more trustable anchors to locate, and then the localization accuracy will be improved. It can be seen that the NRMSE of the proposed method is always the lowest under the different ratio of anchor nodes, especially for less number of anchors. For example, the NRMSE can be less than 0.91 even the ratio of anchor nodes is 0.1 in the simulation scenario. This is because the proposed method can choose more proper anchors than other compared methods.

**Figure 15:**
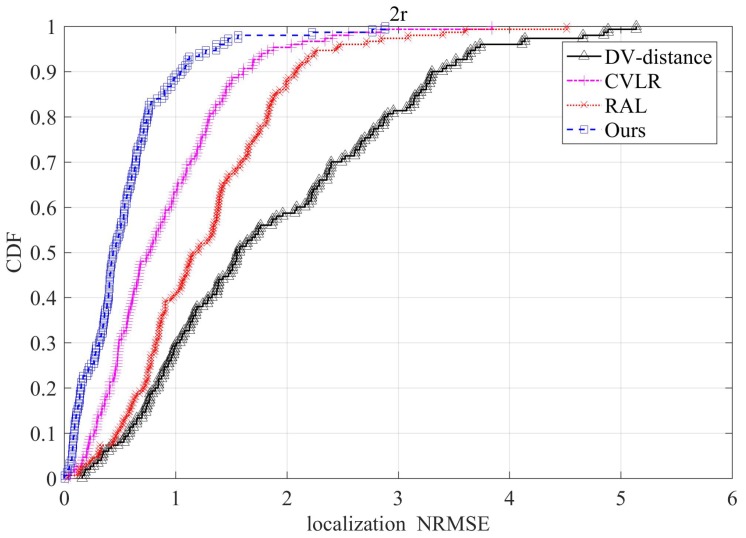
The Cumulative Distribution Function (CDF) of NRMSE under the conditions that node density is 6, and anchor ratio is 0.15.

To further present the performance of these methods, Figure 15 illustrates the Cumulative Distribution Function (CDF) of NRMSE for the compared methods under the conditions that node density is 6, and the ratio of anchor nodes is 0.2. From the figure, it can be seen that by using the proposed method, almost 70% sensor nodes can achieve the localization error less than half of the transmission range *R*. While for CVLR, there are just 50% that can reach the localization accuracy. As for RAL and DV-distance, the two methods have lower localization accuracy. The results validate our proposed method with more details.

### 4.4. Verification of Path Developoing Method

**Figure 16:**
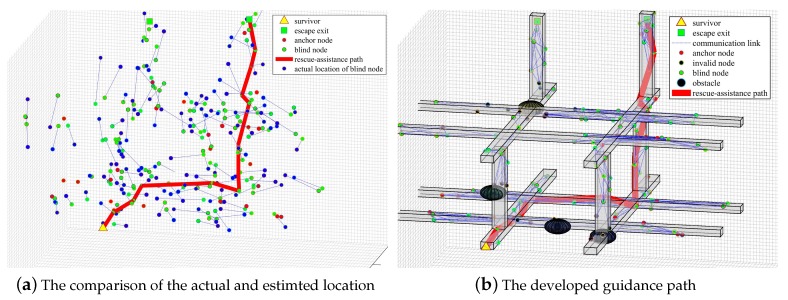
Localization results and guidance paths of the method: (**a**) is the comparison of the actual and estimted location of the blind nodes; (**b**) is the visual result of the escape path search implemented by the algorithm proposed in this paper.

By using the proposed method, the node localization and guidance path results are illustrated in Figure 16. The green rectangle on the top right corner is the escape exit, and the yellow triangle on the bottom left corner is the location of survivors. In Figure 16a, the localization effect is illustrated by comparing the actual locations of the blind nodes, shown as blue dots, and the locations calculated by the proposed method, shown as green dots. The blue dashed lines illustrate the offset of calculated locations to actual locations of nodes. To highlight the effect of node localization, the obstacles are concealed in the sub-figure. It can be seen that most node locations can be calculated accurately. There are some node locations with slightly big offsets. This is because after disasters, in some local areas, the anchor distribution is special sparse, and some nodes cannot find sufficient anchors all the time to locate. The solid pink line is the developed escape path according to the node location. To highlight the effectiveness of the generated path, in Figure 16b, the path is portrayed under the obstacles distribution in the scenario. It can be seen that the developed path is the shortest path from the location of survivors to escape exit without obstacles.

## 5. Conclusions

In this paper, a localization and guidance method is proposed for the wireless sensor network in the underground scenario. The core objective of the method is to generate the optimal escape path for underground trapped survivals in the case of emergency, even in which a collapse has occurred. The proposed method can accelerate the escape procedure and improve the survival probability. On the other hand, the proposed method can provide the rescue-assistance function to ground rescue works by presenting the optimal rescue route. Considering the complex communication environment in the underground scenario, the hybrid channel model is constructed to incorporate the diversity attenuation or penetration characteristics of the wireless signal, such as the fading, multipath, and blocked effect. Then the proposed method can choose the most proper anchor to improve the node localization accuracy. After node localization, the guidance path is generated with the shortest distance and fewer obstacles. The simulation results show that our proposed method can achieve improved localization accuracy and provide the proper guidance path to escape. In further research, the judgment threshold of RSS will be dynamically adjusted to improve the adaptability to the environment change. In the practical application of wireless sensor networks, there may be hidden nodes in the network. Future research will study the influence of such nodes on network localization to improve localization accuracy.

## Figures and Tables

**Table 1:** The symbol notations used in this paper.

Symbol	Description
$P_{tx}$	Transmitting powers of the signal
$P_{rx}$	Receiving powers of the signal
$G_{tx}$	Gain of transmitting antenna
$G_{rx}$	Gain of receiving antenna
$C_{m,n}$	The mode intensity
$E_{m,n}^{eigen}$	The electromagnetic (EM) field distribution
$E_{0}$	The field at the transmitting antenna
$\alpha_{mn}$	The attenuation coefficient
$\beta_{mn}$	The phase shift coefficient
λ	Communication signal wavelength
*d*	Distance between the sender and the receiver
$l_{d}$	Signal propagation loss on the distance *d*
$l_{0}$	Signal propagation loss at the reference distance $d_{0}$
$d_{0}$	Reference distance to calculate propagation loss
$X_{\sigma }$	Gaussian random variable with a zero mean
Hj(fi,d)	The jth channel transfer function
fi	The frequency
*N*	The number of sweep point in every transfer function
*M*	The number of measurements at each point
$P_{rx0}$	Receiving powers of the signal on the reference $d_{0}$
*C*	Threshold value of the number of signals that pass-through obstacles
$W_{a}$	The attenuation effect caused by the number of obstacles
$F_{a}$	The obstacle attenuation factor
*B*	The piecewise attenuation factor caused by obstacles
$l_{f}$	The reference propagation loss between nodes *d*
$l_{r}$	The measured propagation loss between nodes *d*
$\eta_{1},\eta_{2}$	The piecewise condition parameter of hybrid model *d*
$R_{i}$	The location of anchor *i*
$x_{i}$	The x-coordinate of anchor *i*
$y_{i}$	The y-coordinate of anchor *i*
$z_{i}$	The z-coordinate of anchor *i*
$X_{n}$	The location of node *n*
$x_{n}$	The x-coordinate of blind node *n*
$y_{n}$	The y-coordinate of blind node *n*
$z_{n}$	The z-coordinate of blind node *n*
$d_{i}$	The distance between anchor *i* and node *n*
$R_{T}$	The trustable anchor
$R_{T,i}$	The *i*th location of trustable anchors close to node *n*
$A_{R}$	The set of candidate anchors
i,j	The indices of the anchors
$i^{*},j^{*}$	The indices of the chosen anchors
$X_{n,k}$	The *k*th candidate location of node *n*
*k*	The index of the candidate locations of node *n*
$s_{s}$	The source node of guidance path
$s_{t}$	The target node of guidance path
*h*	The number of hops
$P_{st}^{h}$	The matrix saving all found paths
$D_{st}^{h}$	The matrix saving the distances of all found paths
$p^{h}$	The guidance path
$p_{h}$	One of the paths
$d_{sum}$	The distance an path $p_{h}$

**Table 2:** Simulation parameter table.

Parameter	Value
Transmit power	−5 dBm
Antenna gain	2 dBi
Signal frequency	2.4 GHz
Scene size	80 m × 80 m × 60 m
Tunnel size	width: 2 m/height: 2 m
Node density	4/5/6/7/8/9/10
Anchor node ratio	0.10/0.15/0.20/0.25/0.30/0.35/0.40
